# Structural analysis of TRIM family PRYSPRY domains and its implications for E3-ligand design

**DOI:** 10.1016/j.yjsbx.2025.100134

**Published:** 2025-07-30

**Authors:** Rezart Zhubi, Apirat Chaikuad, Christian J. Muñoz Sosa, Andreas C. Joerger, Stefan Knapp

**Affiliations:** a Institute of Pharmaceutical Chemistry, Goethe University, Max-von-Laue-Str. 9, 60438 Frankfurt am Main, Germany; b Structural Genomics Consortium (SGC), Buchmann Institute for Life Sciences, Max-von-Laue-Str. 15, 60438 Frankfurt am Main, Germany

## Abstract

Tripartite motif (TRIM) proteins constitute one of the largest subfamilies of RING-type E3 ubiquitin ligases and are attractive targets for the development of novel degraders that exploit the ubiquitin–proteasome pathway. More than half of all TRIM family members contain a PRYSPRY domain, a potentially druggable protein interaction module, located in their C-terminal region. Here, we have determined crystal structures of the PRYSPRY domains from nine TRIM family proteins: TRIM1 (MID2), TRIM9, TRIM10, TRIM11, TRIM15, TRIM16, TRIM18 (MID1), TRIM36, and TRIM67. These structures reveal conservation of the overall β-sandwich topology, despite low sequence conservation, with a unique subdomain swap observed in TRIM11. Significant variations were found in the loops flanking the canonical substrate-binding site, which modulate the shape and electrostatic properties of the binding pocket, hinting at substantial differences in substrate specificity and binding modes among family members. TRIM36 features a unique structural motif between the canonical β-strands 2 and 3, leading to the formation of a dimer, with the canonical substrate-binding site partially occluded by the dimerization motif. In addition, we mapped the locations of missense mutations in MID1 associated with X-linked Opitz syndrome, suggesting that some of these mutations impair the conformational stability of the protein. Taken together, our data provide intriguing insights into the structural and functional divergence of TRIM family PRYSPRY domains, their potential druggability and substrate recognition, and the challenges of ligand design.

## Highlights

• Crystal structures of the PRYSPRY domain of nine TRIM-family proteins determined.
• Structural and functional divergence of TRIM-family PRYSPRY domains.
• Unique subdomain swapping in TRIM11 and dimerization motif in TRIM36.
• Opitz-syndrome-associated mutations in MID1 PRYSPRY domain mapped.
• Potential druggability and challenges in ligand design assessed.

## Graphical abstract

**Figure:**
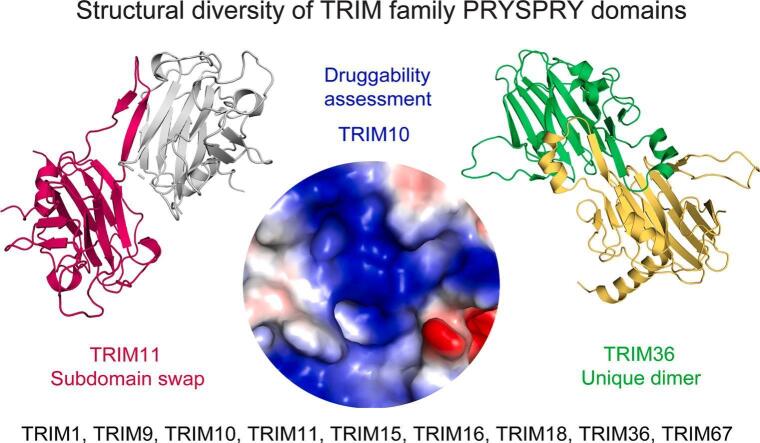

## Introduction

**Fig. 1:**
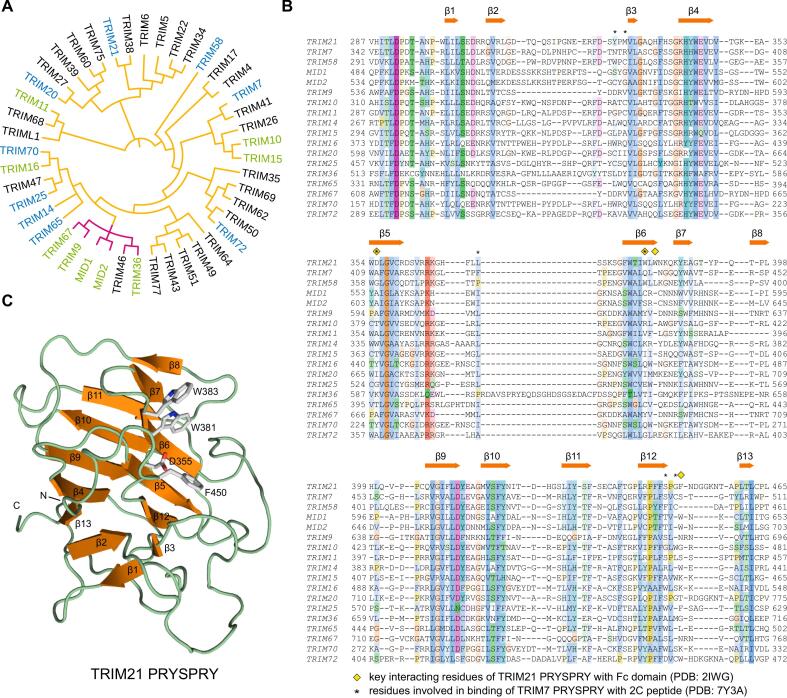
Sequence, structure, and evolutionary relationships of TRIM E3 ligase PRYSPRY domains. (A) Phylogenetic tree of TRIM family PRYSPRY domains. The PRYSPRY structures of TRIM family members highlighted in blue have been determined previously, while the structures of those highlighted in green have been determined in this study. Orange branches indicate subfamily IV, and magenta branches indicate subfamily I of the TRIM family. The tree was generated using the Interactive Tree Of Life (iTOL) online tool at https://itol.embl.de/ (Letunic and Bork, 2021). (B) Structure-based sequence alignment of human PRYSPRY domains with available structures in the Protein Data Bank. Residues are colored by type and sequence conservation. Secondary structure elements shown above the alignment are based on the TRIM21 structure. Key residues of TRIM21 interacting with antibody IgG:Fc (PDB: 2IWG) and of TRIM7 interacting with the 2C peptide (PDB: 7Y3C) are highlighted. (C) Cartoon representation of the overall structure of the human TRIM21 PRYSPRY domain (PDB: 2IWG). Side chains of key residues involved in binding of antibody IgG:Fc are shown as gray stick models.

The tripartite motif (TRIM) proteins form one of the largest subfamilies of RING-type E3 ubiquitin ligases, comprising more than 80 members. TRIM family members typically contain a RING domain usually catalyzing ubiquitin transfer; one or two B-box domains mediating protein–protein interactions; and a large coiled-coil domain region, which is involved in self-association or oligomerization with other TRIM E3-ligase proteins (Fiorentini et al., 2020, Ozato et al., 2008). The C-terminal region, responsible for substrate recognition, varies among different family members, and the TRIM family has been divided into 11 distinct subgroups based on the composition of this region (Chaikuad et al., 2022, Hatakeyama, 2011). About two-thirds of human TRIM proteins feature a PRYSPRY domain, also known as the B30.2 domain, in their C-terminal region. The SPRY domain is an evolutionarily conserved protein–protein interaction motif, first identified in dual-specificity kinase splA of *Dictyostelium discoideum* and ryanodine receptor subtypes (Ponting et al., 1997). The broader SPRY family can be divided into two subfamilies: the B30.2 (or PRYSPRY) subfamily, with a length of ∼ 200 amino acid residues, that includes an additional N-terminal PRY motif, and the SPRY-only class, which is ∼ 140 amino acids long. The first human PRYSPRY domain structure revealed that the overall fold of this protein domain consists of a 7-stranded and a 6-stranded antiparallel β-sheet forming a β-sandwich (Fig. 1). The N-terminal PRY subdomain comprises three β-strands, followed by the SPRY motif with 10 β-strands, all connected by loops of varying lengths (Grütter et al., 2006). More than 40 TRIMs contain a SPRY domain at their C-terminus, most of which belong to the PRYSPRY subfamily (D'Cruz et al., 2013, Hatakeyama, 2017). However, these domains exhibit low sequence homology, consistent with diverse interaction partners of the SPRY domains. The structures of TRIM21-IgG Fc, TRIM65-MDA5, and TRIM7-peptide complexes revealed distinct substrate recognition mechanisms, involving loops, β-turns, helical motifs, and C-terminal peptides (James et al., 2007, Kato et al., 2021, Liang et al., 2022). SPRY domains are also found in E3 ligases with a different overall domain organization than the TRIM family, including SPRY domain-containing SOCS box-adapting proteins, where the domain contains a C-terminal helical extension for interaction with elongin B/C adaptors in cullin-RING E3 ubiquitin ligase complexes (Cai and Yang, 2016, Woo et al., 2006), but also in proteins without E3 ligase function.

TRIM proteins are involved in diverse cellular functions, including antiviral activity, cell differentiation, innate immunity, autophagy, and carcinogenesis (Chabot et al., 2025, Hatakeyama, 2017, Meroni and Diez-Roux, 2005, Wang and Hur, 2021). Some of the PRYSPRY-containing TRIM family proteins structurally characterized in this study, for example, modulate innate immune signaling complexes (e.g., TRIM10, TRIM11, or TRIM15) (Chabot et al., 2025, Kong et al., 2023), promote apoptosis via inhibition of the Wnt/β-catenin signaling pathway (TRIM36) (Zhao et al., 2022), regulate cell-cycle progression via microtubule association (TRIM36), or play a role in neuronal development and synaptic regulation (TRIM9 and TRIM67) (Bouron and Fauvarque, 2022, Menon et al., 2015). Moreover, TRIM16 has been reported to act as a scaffolding protein that facilitates autophagic degradation of protein aggregates (Jena et al., 2018).

Mutations within the PRYSPRY domains of various TRIMs have been linked to susceptibility to certain diseases. For example, mutations in TRIM20/pyrin are associated with familial Mediterranean fever (Chae et al., 2003), while mutations occurring in TRIM18/MID1 have been associated with X-linked Opitz G/BBB syndrome (Baldini et al., 2020, Trockenbacher et al., 2001). Furthermore, TRIM21 acts as an autoantigen in diseases such as rheumatoid arthritis, systemic lupus erythematosus, and Sjögren’s syndrome (Ishii et al., 2003, Jones et al., 2021).

TRIM family proteins have gained increasing interest as potential therapeutic targets and as attractive alternative E3 ubiquitin ligases to the established cereblon (CRBN) and von Hippel-Lindau (VHL) for targeted protein degradation via the ubiquitin–proteasome pathway, especially those that are exclusively expressed in a specific tissue (Hartmann, 2024). In particular, TRIM21 has been shown to be a versatile E3 ligase for the degradation of cellular proteins using the antibody-based TRIM-Away technology (Clift et al., 2017, Clift et al., 2018). TRIM-Away can rapidly degrade not only cellular proteins but also large pathogens such as viruses (Yang et al., 2024). Furthermore, ligands have been identified that act as molecular glues to promote the degradation of large assemblies, such as the nuclear pore complex (Lu et al., 2024). However, a recent family-wide study providing a detailed structure–function analysis of the ubiquitin E3 ligase activity revealed that some RING domains of TRIM family members are structurally divergent, disrupting their ability to catalyze ubiquitin transfer. These should therefore be considered “pseudoligases” (Dudley-Fraser et al., 2025).

Recently, the structure of the human TRIM58 PRYSPRY domain in complex with a small molecule, TRIM-473, bound in the canonical binding site was reported, providing unique insights into substrate binding and a starting point for the development of tissue-specific degraders (Hoegenauer et al., 2023). In addition, the structure of TRIM7 with a peptidomimetic ligand has been published, highlighting the potential druggability of TRIM family PRYSPRY domains but also its challenges (Munoz Sosa et al., 2024); and more recently, a covalent ligand of TRIM25 has been identified (McPhie et al., 2025). Several ligands for TRIM21 have been described, including a potent DEL (DNA-encoded library) hit (Li et al., 2025), the drug suramin (Kim et al., 2025a), and molecular glue degraders (Cheng et al., 2025). Moreover, a recent comprehensive study from our group on chemical fragments and their binding modes (Kim et al., 2025b) highlights the excellent druggability of this PRYSPRY domain.

Here, we performed a systematic structural characterization of the PRYSPRY domain from nine additional TRIM family members, revealing conservation of the overall fold but, importantly, also unique structural features in some family members and variations in the length and composition of the loops lining the putative substrate-binding sites. These features modulate the shape and electrostatics of the canonical binding site, suggesting both distinct substrate-binding modes and differences in their potential druggability with small molecules.

## Results

### Overall structure of TRIM family PRYSPRY domains

**Fig. 2:**
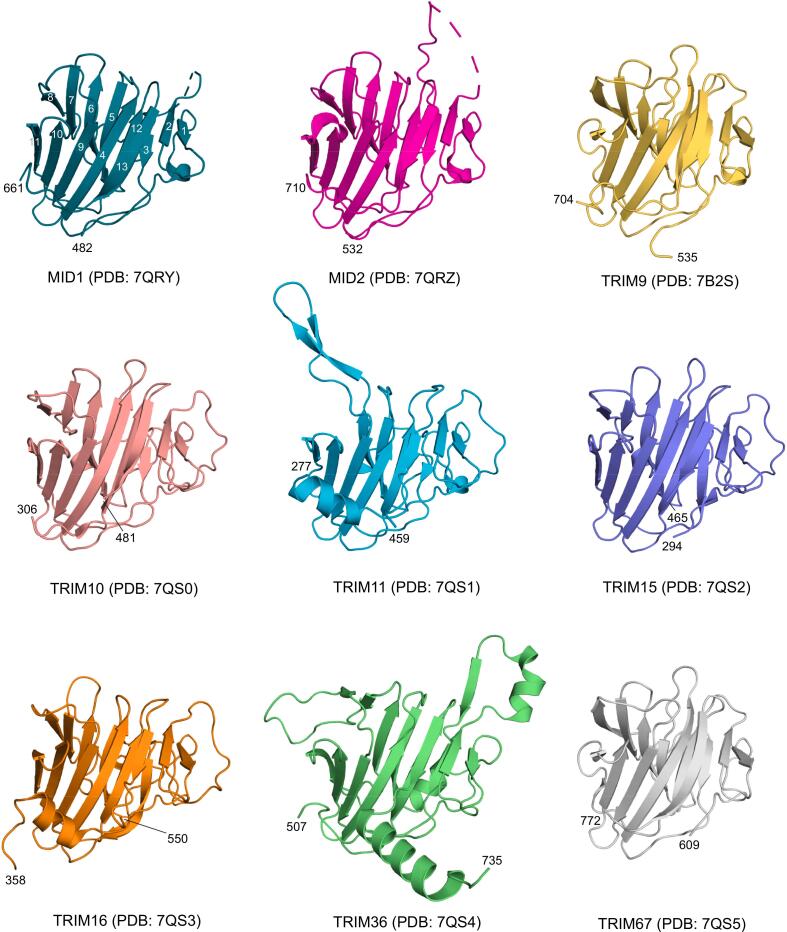
Overall fold of the structures of human TRIM PRYSPRY domains determined in this study. The N- and C-terminal residues of each structure are labeled. The structures of TRIM11 and TRIM16 feature an N-terminal helical extension, while the structure of the TRIM36 domain includes an additional C-terminal helix. The most striking overall structural differences within the PRYSPRY domain itself are large loop insertions in MID1/2 (either partly or largely disordered), a unique insertion in TRIM36 involved in homodimerization, and a displacement of the β-strand 7,8 segment in TRIM11 (See main text for further details).

**Fig. 3:**
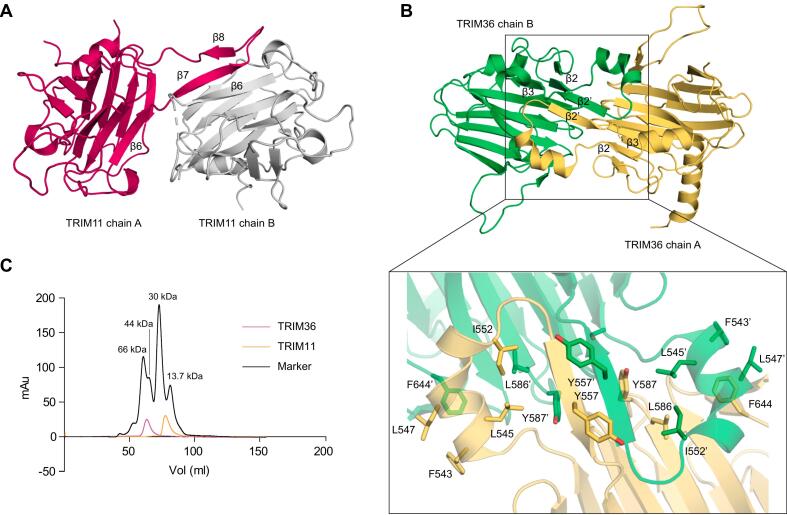
Structural differences in TRIM11 and TRIM36 PRYSPRY domains. (A) Asymmetric unit of the TRIM11 PRYSPRY structure showing subdomain swapping. β-strands 7 and 8 of chain A (shown in dark red) complete the canonical PRYSPRY fold in chain B (shown in gray), whereas the equivalent structural elements of chain B are disordered. (B) Symmetrical dimer in the crystal structure of the TRIM36 PRYSPRY domain. The dimer interface is formed via the insertion connecting the canonical β-strands 2 and 3. (C) Size-exclusion chromatography of the TRIM11 and TRIM36 PRYSPRY domain on a Superdex-75 column compared with the elution profiles of marker proteins. TRIM11 eluted at a retention time corresponding to a monomer (orange trace; theoretical MW of the monomer = 20.9 kDa), whereas TRIM36 eluted at a retention time corresponding to a dimer, in agreement with the structural data (magenta trace; theoretical MW of the dimer = 52.4 kDa). Marker proteins used: bovine serum albumin (MW = 66 kDa; Sigma-Aldrich), hen egg white ovalbumin (MW = 44 kDa, Sigma-Aldrich), bovine erythrocyte carbonic anhydrase (MW = 30 kDa, MP Biomedicals), bovine ribonuclease A (MW = 13.7 kDa, Sigma-Aldrich).

To extend the portfolio of available TRIM family PRYSPRY domain structures, we cloned different domain variants from 18 family members into bacterial expression vectors. For 15 of these family members, we were able to produce sufficient soluble protein for crystallization trials (**Supporting Table S1**). This enabled us to determine the crystal structures of nine different TRIM family PRYSPRY domains at a resolution ranging from 1.5 to 2.3 Å: TRIM1 (MID2), TRIM9, TRIM10, TRIM11, TRIM15, TRIM16, TRIM18 (MID1), TRIM36, and TRIM67 (**Supporting Table S2**). The structure of MID2 was determined in two different crystal forms. Except for TRIM36 (isomorphous replacement using a mercury derivative), all structures were solved by molecular replacement. All characterized family members shared the same overall PRYSPRY twisted β-sandwich fold consisting of a 7-stranded and a 6-stranded antiparallel β-sheet (Fig. 2). A notable exception was the TRIM11 structure, in which a unique subdomain swap was observed. The crystal lattice contained two protein molecules in the asymmetric unit. In chain A, β-strands 7 and 8, usually located at the edge of the concave 6-stranded β-sheet, were displaced and interacted with the corresponding region in chain B, forming a hybrid β-sandwich (Fig. 3**A**). The equivalent β-strands in chain B, however, did not interact with chain A but were disordered, resulting in an asymmetrical dimer within the asymmetric unit. These observations raised the question of whether this domain might form a symmetrical subdomain-swapped dimer in solution. However, gel filtration analysis showed that the isolated TRIM11 PRYSPRY domain is largely monomeric in solution (Fig. 3**C**). Nevertheless, the observed subdomain swap in the crystal indicates an increased structural plasticity of this region of the TRIM11 PRYSPRY domain that was not seen for other family members. In this context, it is also noteworthy that the length and relative orientation of these two β-strands (especially strand 8), as well as the connecting loop, differ significantly among family members. These two β-strands are particularly long in TRIM9/67 and much shorter in TRIM10/15 and TRIM36 (Fig. 1**B** and Fig. 2).

There are significant variations in the length, conformation, and amino acid composition of the loops connecting the β-strands. For example, we observed a unique 11-residue deletion between β-strands 2 and 3 in the phylogenetically more closely related family members TRIM9 and TRIM67 (Fig. 1**A/B** and Fig. 2). Conversely, the TRIM36 PRYSPRY domain has characteristic loop insertions, which also affects its oligomerization state. The crystal lattice of the TRIM36 PRYSPRY domain contained four molecules in the asymmetric unit, forming two essentially identical symmetrical dimers. This observation is consistent with the TRIM36 domain eluting in gel filtration with a Superdex-75 column at a retention time corresponding to a dimer (Fig. 3**C**). The dimer interface is formed by a helix-turn-strand insertion between the canonical β-strands 2 and 3 of the PRY subdomain (Fig. 2). The additional β-strand, β2′, self-assembled into a two-stranded antiparallel intermolecular β-sheet, and Phe543, Leu545, Leu547, and Ile552, located on the helix, form hydrophobic packing interactions with the adjacent monomer (Fig. 3**B**). The total dimer interface area is 1803 Å^2^ for chains A and B, and 1875 Å^2^ for chains C and D. The same PRYSPRY dimer interface was also predicted in the AlphaFold3 (Abramson et al., 2024) model of the full-length protein of TRIM36, and the related TRIM46 homolog, in addition to the typical dimerization via the coiled-coil domain (see Fig. 1**A** and **Supporting Information Fig. S1**). Interestingly, PISA analysis (Krissinel and Henrick, 2007) of the PRYSPRY crystal structure suggested a tetramer (dimer of dimers contained in the asymmetric unit) as a stable assembly in solution.

An unusually large insertion was observed between the canonical β-strands 5 and 6, featuring an additional helix and a long disordered segment. Such a large loop insertion with predominantly random coil structure at this position was also predicted for the closely related TRIM46, whereas TRIM26 features an even larger insertion between β-strands 4 and 5, which is mainly composed of negatively charged residues. Another noteworthy feature of the TRIM36 structure is a unique helical extension at the C-terminus.

### Analysis of the putative substrate-binding pocket

The reported structures of TRIM PRYSPRY domains with natural substrates map the binding sites to a common region on top of the 6-stranded β-sheet, with the exact nature of this site modulated by the surrounding loops (James et al., 2007, Kato et al., 2021, Liang et al., 2022). In the case of TRIM21, there are two distinct subpockets for interaction with different parts of the antibody in the complex with IgG Fc (James et al., 2007). A systematic analysis of this canonical substrate-binding region in the PRYSPRY structures determined in this study revealed significant variations and distinct surface properties across different family members, providing intriguing insights into the divergent evolution of TRIM family members. This canonical binding site was accessible to a varying degree in the domains crystallized here, except for TRIM36. In this case, dimerization via the insertion between the canonical β-strands 2 and 3, as described above (Fig. 3**B**), effectively occluded much of the putative substrate-binding site.

#### Shallow surface in the canonical substrate-binding site of TRIM9 and TRIM67

**Fig. 4:**
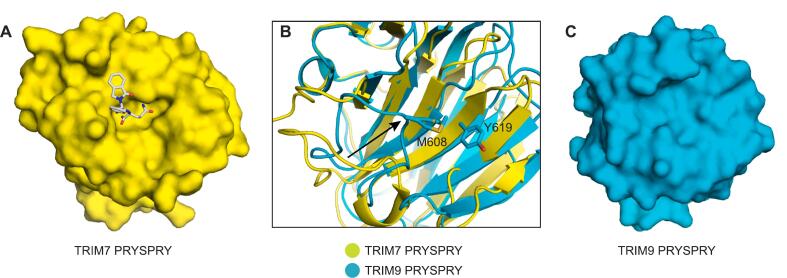
Comparison of the canonical substrate-binding site in TRIM7 PRYSPRY with the corresponding region in TRIM9. (A) Crystal structure of human TRIM7 PRYSPRY (PDB entry 8R5C) shown as a surface representation. The bound peptidomimetic ligand is shown as a grey stick model. (B) Superimposition of the structures of TRIM7 and TRIM9, highlighting the different orientation of the β5/β6 loop in TRIM9, which collapsed onto the binding pocket via Met608-mediated interactions. The black arrow indicates the relative movement of this loop in TRIM9. (C) Molecular surface of the putative substrate-binding site in TRIM9, revealing a relatively shallow binding surface compared to TRIM7.

Compared with the recently determined structures of TRIM7 PRYSPRY with a peptide substrate or a peptidomimetic ligand (Fig. 4**A**) (Liang et al., 2022, Munoz Sosa et al., 2024), the putative substrate-binding site in the structure of the two phylogenetically closely related family members TRIM9 and TRIM67 was much shallower (Fig. 4**C**). This was largely, but not only, due to a distinct orientation of the β5/β6 loop. This loop lines the pocket in the TRIM7-ligand complex but folds back onto the β-sandwich in TRIM9 and TRIM67 via a methionine that is unique to those two family members **(**Fig. 1**A,** Fig. 4**B)**. In TRIM9, this methionine, Met608, packs against Tyr619 on β-strand 6. The equivalent interacting residues in TRIM67 exemplifying this loop collapse are Met680 and Tyr691. That this was seen in both the TRIM9 and TRIM67 structures suggests it is an intrinsic property of this loop in these two closely related homologs, rather than being induced by crystal packing.

#### MID1 and MID2

**Fig. 5:**
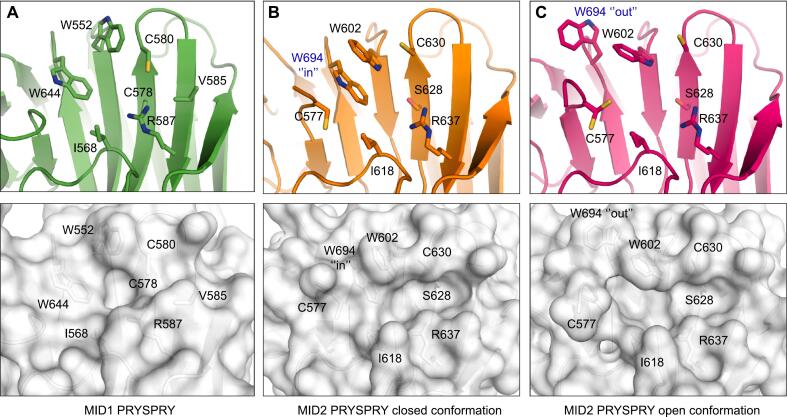
Putative substrate-binding pocket of the MID1 and MID2 PRYSPRY domains. The top panel shows ribbon diagrams of each structure, with the side chains of key residues lining the pocket shown as stick models. In the lower panel, the structures are shown as surface representations in the same orientation as above. (A) MID1, (B) MID2, crystal form in space group P2_1_2_1_2_1_, chain B (open conformation), (C) MID2, crystal form in space group P2_1_2_1_2_1_, chain A (closed conformation). Comparison of the two molecules in the asymmetric unit in this MID2 structure showed a different orientation of the side chain of Trp694, which modulates the size of the putative substrate-binding pocket, indicating a certain degree of structural plasticity.

A characteristic feature of the putative substrate-binding site in the closely related homologs MID1 and MID2 is an arginine located at the C-terminus of β-strand 7 (R587 in MID1 and R637 in MID2) that projects its guanidinium group toward the center of the cavity and two neighboring tryptophan residues lining the pocket (Fig. 5). The orientation of the side chain of the two tryptophan residues in MID2, Trp602 and Trp694, showed an intriguing variation in the crystal structures. The MID2 domain was solved in two different crystal forms. In the C222_1_ crystal form, which contains one protein molecule per asymmetric unit, the side chains of Trp602 and Trp694 engage in π-stacking interactions. In the other crystal form, in P2_1_2_1_2_1_, which contains two molecules per asymmetric unit, an alternative orientation was observed in one of the two chains, with the side chain of Trp694 swinging out of the pocket (Fig. 5**B/C**). This alternative conformation substantially increases the size and depth of the pocket, leaving the side chain of the neighboring Trp602 accessible for packing interactions with a potential substrate. CASTp analysis (Tian et al., 2018) detected a pocket with a solvent-accessible (SA) volume of 56 Å^3^ in chain A but only a much smaller subpocket at this site in chain B (SA volume = 1.8 Å^3^). It remains to be seen whether this more open state is sufficiently populated in solution to provide a targetable interaction hotspot at Trp602.

#### TRIM10 and TRIM15

**Fig. 6:**
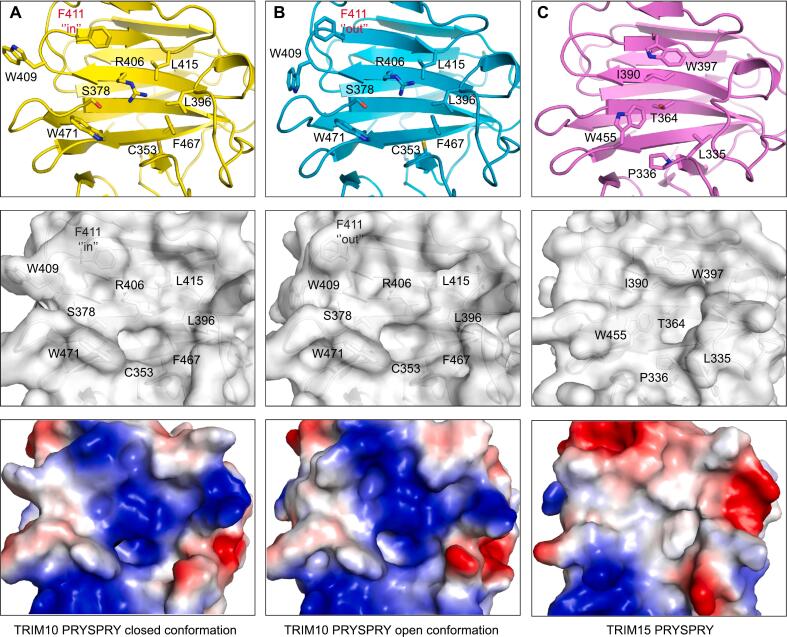
Putative substrate-binding pocket in TRIM10 and TRIM15. The top panel shows ribbon diagrams of each structure, with the side chains of key residues lining the pocket highlighted as stick models. The middle panel displays semi-transparent surface representations in the same orientation, and the bottom panel shows the molecular surface colored according to its electrostatic potential (blue, positive electrostatic potential; red, negative electrostatic potential). (A) TRIM10 PRYSPRY domain chain A. (B) TRIM10 PRYSPRY domain, chain C, showing a more open pocket due to a flip of the Phe411 side chain. (C) TRIM15 PRYSPRY domain.

As observed with MID2, the crystal structure of TRIM10 also provided insights into the conformational equilibrium of the loops lining the putative substrate-binding pocket. This structure was determined in a crystal form with three molecules in the asymmetric unit, and comparison of the different chains revealed a varying conformation of the Phe411 side chain, which modulates the shape of the binding site (Fig. 6**A/B**). An arginine at the center of the pocket, Arg406 on β-strand 6, is positioned in such a way that its guanidinium group is poised for both electrostatic and cation-π interactions with potential substrates or small-molecule ligands. This central arginine is flanked by several hydrophobic residues, including a tryptophan (Trp471 at the C terminus of β-strand 12) and the above-mentioned Phe411. Due to the generally shallow nature of the PRYSPRY substrate-binding pockets, small conformational changes at the periphery can significantly affect the results from common druggability predictors or pocket size calculations. For example, CASTp (Tian et al., 2018) identified a pocket with a solvent-accessible area of 142 Å^2^ and a solvent-accessible volume of 150 Å^3^ for the putative substrate-binding site in chain C, but detected only a very small pocket at the same site in chains A and B (solvent-accessible volumes of 4 and 8 Å^3^, respectively).

In the TRIM15 crystal structure, a reasonably pronounced pocket was observed, with a solvent-accessible area of 83 Å^2^ and a solvent-accessible volume of 46 Å^3^ (Fig. 6**C**). TRIM15 lacks the arginine at the center of the pocket seen in TRIM10, which is replaced by an isoleucine (Ile390), resulting in a substantially more hydrophobic pocket. There are two tryptophan side chains on opposite sides of the binding pocket (Trp397 and Trp455) that provide hydrophobic docking surfaces. Trp397 is conserved in TRIM10 but adopts a different conformation in TRIM15, pointing toward the center of the pocket rather than outward. For the TRIM16 structure, CASTp failed to detect a pocket in the canonical substrate-binding site due to its shallow nature.

### Location of Opitz-syndrome-associated mutations in MID1 PRYSPRY

**Fig. 7:**
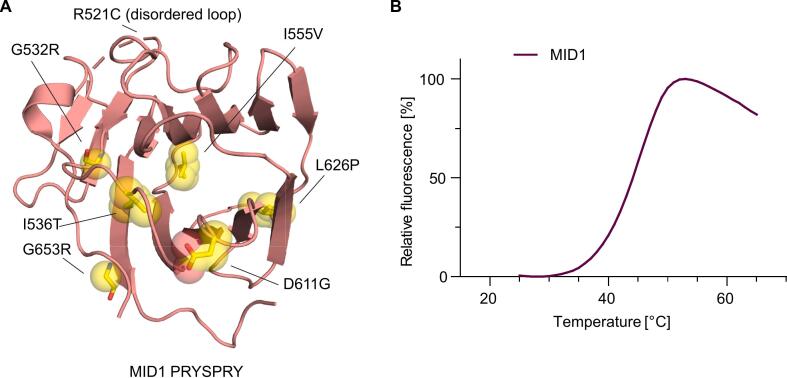
Location of disease-related mutations in MID1 PRYSPRY. (A) Location of mutations in the MID1 PRYSPRY domain associated with Opitz G/BBB syndrome. (B) Thermal unfolding curve of the MID1 PRYSPRY domain determined by differential scanning fluorimetry.

MID1 is mutated in X-linked Opitz G/BBB syndrome, a rare genetic disorder that results in malformations along the midline of the body, including hypertelorism, hypospadias, and laryngo-tracheo-esophageal defects (Baldini et al., 2020, Trockenbacher et al., 2001). Many of the reported mutations map to the PRYSPRY domain (Baldini et al., 2020, Fontanella et al., 2008). In addition to nonsense, frameshift, and splice-site mutations, several missense mutations have been identified, distributed across the entire domain (Fig. 7**A**).

Some of these, such as I536T and I555V, are large-to-small substitutions that potentially create internal cavities in the hydrophobic core of the β-sandwich, thereby reducing the thermodynamic stability of the domain. Similar mutations in the DNA-binding domain of the tumor suppressor p53 have been associated with stability loss and global unfolding at body temperature (Balourdas et al., 2024, Joerger et al., 2006). Like p53, the MID1 PRYSPRY domain is only moderately stable (DSF *T*_m_ of 47 °C; Fig. 7**B**), so it is also susceptible to global unfolding by cavity-creating mutations. In the case of a conservative mutation such as I555V, where only a single methyl group is lost, however, the stability loss may be only moderate. The L626P mutation also perturbs the hydrophobic core of the β-sandwich and, being located in the middle of β-strand 11, also disrupts interstrand hydrogen bonds. The small-to-large mutation G532R introduces severe steric clashes and would bury a positive charge within a cluster of hydrophobic side chains, including Phe486 and Trp543. Therefore, this mutation is also likely to severely compromise the folding of the domain.

Other mutations affect residues with surface-exposed side chains and may directly alter functional interfaces. The D611G mutation, for example, is located on the short loop connecting β-strands 9 and 10. This mutation results in the loss of a stabilizing hydrogen bond between the carboxylate group of Asp611 and the backbone nitrogen of Gly539 in the β3/β4 turn. In addition, it introduces increased flexibility due to mutation to a glycine. Besides the above-mentioned G532R mutation, there is another Opitz-syndrome-associated mutation of a glycine to an arginine, G653R, which is located at the C-terminus of the last β-strand. In this instance, however, the mutation results in a surface-exposed arginine that induces an altered surface complementarity, which may be functionally relevant. In another case, a surface-exposed arginine is mutated to a cysteine, R521C (Ji et al., 2014). Here, the mutation site is located in the disordered region of the loop between β-strands 2 and 3, at the edge of the canonical substrate-binding site.

## Discussion

We have determined crystal structures of the PRYSPRY domain from nine members of the TRIM family. Despite low sequence conservation, they mostly featured the same β-sandwich fold, composed of a 6-stranded and a 7-stranded antiparallel β-sheet. An interesting variation was observed for TRIM11, however, where two β-strands of the 6-stranded β-sheet showed increased structural plasticity, resulting in this segment either being disordered or forming a hybrid sheet with a neighboring molecule. While this assembly is stabilized by crystal packing, it nonetheless reveals the intrinsic flexibility of the swapped β-hairpin motif in this family member. This is somewhat reminiscent of the structure of type 1 ryanodine receptor (PDB entry 7TZC), which contains three SPRY domain modules, where the equivalent β-hairpin segment of the third SPRY module is displaced and extends a β-sheet of another SPRY domain within the same polypeptide chain (Melville et al., 2022).

Substantial structural differences were observed in the loops connecting the β-strands between different family members, featuring, for example, a unique dimerization motif in TRIM36 that results in the formation of stable dimers in solution. Dimerization and higher-order assembly of TRIM proteins are usually mediated by the coiled-coil domain, not the PRYSPRY domain. In the case of TRIM72, for example, this arrangement places two PRYSPRY domains in close proximity on the same face of the dimer, but with their positively charged substrate-binding surfaces oriented away from each other. This orientation enables cooperative binding to phosphatidylserine-enriched membranes, a mechanism suggested to be essential for the membrane repair function of TRIM72 (Ma et al., 2023, Park et al., 2023). A symmetrical dimeric assembly of a PRYSPRY domain has been observed in the apo structure of TRIM58 (PDB entry 8PD4). In that case, the interactions are mediated via an N-terminal extension that includes a helical segment of the coiled-coil domain around Leu264, which wraps around the PRYSPRY domain of a neighboring molecule. However, this is most likely a crystal packing artifact of the domain construct used, rather than a stable assembly in solution. Such an assembly would block the ligand binding observed, using the same construct, in PDB entry 8PD6 (Hoegenauer et al., 2023), and is also incompatible with the expected spatial distance of the interacting segments upon coiled-coil-mediated dimerization in the full-length protein. The closed PRYSPRY dimer seen for TRIM36 − and predicted for TRIM46 − is therefore unique, and intriguing. It will be particularly interesting to determine whether this assembly is of functional relevance, for example by facilitating specific scaffolding roles or mediating microtubule interactions.

**Fig. 8:**
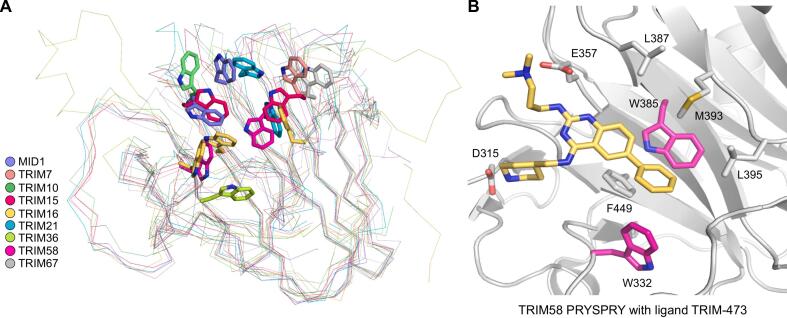
Location of tryptophan residues in the canonical substrate-binding site of PRYSPRY domains. (A) Permutation of tryptophan residues in the putative substrate-binding pocket across different TRIM PRYSPRY domains. (B) Binding mode of TRIM58 ligand TRIM-473 (PDB: 8PD6; (Hoegenauer et al., 2023)). The two tryptophan side chains packing against the phenyl moiety of the ligand (shown as a yellow stick model) are highlighted in magenta.

The observed differences in the loops connecting the β-strands also affect those flanking the canonical substrate-binding site, resulting in variations in the size, shape, and polarity of this potential interaction surface, which reflects the functional divergence of the TRIM family PRYSPRY domain. The overall structural divergence of the canonical substrate-binding site in the TRIM family PRYSPRY domain is nicely illustrated by the permutation of tryptophan residues, potential interaction hotspots, across the binding site in the different family members (Fig. 8**A**). The recently reported TRIM58 ligand, TRIM-473, is sandwiched between two tryptophan side chains in the binding site (Fig. 8**B**). A recent high-throughput crystallography-based fragment screening of TRIM21 identified the two tryptophan residues located in the substrate-binding site as interaction hotspots, with many fragments packing against their indole side chain (Kim et al., 2025b). The electrostatic nature of the canonical binding site varied significantly in the newly determined PRYSPRY structures. Some feature an arginine facing the central region of the pocket, though contributed by different structural segments (e.g. MID1, MID2, or TRIM10; Fig. 5, Fig. 6), indicating a potential interaction with side-chain carboxylates or C-terminal peptides as observed for the TRIM7-2C complex (Liang et al., 2022). In contrast, others had a largely hydrophobic center of the pocket (e.g. TRIM15, Fig. 6**C**).

The variations in the putative substrate-binding site across different PRYSPRY domains have important implications for drug design, highlighting specific challenges in targeting some of the family members and revealing significant differences in druggability within the family. The binding site generally tends to have a shallow topology, and conventional pocket analysis software failed to detect a sizeable pocket at this site in some cases, as discussed above for TRIM16. This does not, per se, mean that small-molecule ligands cannot be identified, as demonstrated by the recent discovery of ligands for TRIM58 (Hoegenauer et al., 2023). However, developing high-affinity binders is expected to be particularly challenging for those family members. In some cases, such as MID2, it may be possible to target transient protein states with more open pockets, provided these states are sufficiently populated in solution. Such cryptic sites may also exist in other PRYSPRY members.

It is clear that even relatively small differences in the conformational state of the loops flanking the putative substrate-binding site can have a significant effect on the surface complementarity of this region and, consequently, its druggability. Computational drug discovery efforts should, therefore, take a sufficient degree of conformational plasticity into account, for example, via ensemble docking from MD simulations, to also sample transient protein states.

In summary, our structural data provide a platform for future drug discovery efforts but, importantly, also hint at significant challenges in developing potent small-molecule binders targeting the canonical binding site, especially in cases of particularly shallow surfaces and those partly or largely occluded by either loop collapse (TRIM9/67) or dimerization (TRIM36).

## Materials and methods

### Recombinant protein production and crystallization of TRIM family PRYSPRY domains

The cDNAs of the PRYSPRY domains of human MID1 (TRIM18), MID2 (TRIM1), TRIM9, TRIM11, TRIM15, TRIM16, and TRIM67 were subcloned into a pGTVL2 vector; TRIM10 was subcloned into pSUMO-Lic, and TRIM36 was subcloned into a pNIC-CTH0 vector. The recombinant proteins were accordingly expressed as either His_6_-GST, His_6_-SUMO, or His_6_ fusion in *Escherichia coli* Rosetta strain cultured in TB media at 37 °C until an OD_600_ of 1.6–1.8 was reached. At this point, the cultures were cooled to 18 °C, and at an OD_600_ of 2.6–2.8, gene expression was induced with 0.5 mM IPTG overnight.

The recombinant proteins were first purified by Ni^2+^-affinity chromatography. The His_6_-GST and His_6_ tags were removed by TEV protease cleavage, whereas the His_6_-SUMO tag was cleaved by sentrin-specific protease 1 treatment. The cleaved proteins were then separated by passing them through Ni^2+^ beads and further purified by size-exclusion chromatography using a HiLoad 26/600 Superdex-75 pg column (Cytiva) with the buffer containing 20 mM HEPES pH 7.5, 100 mM NaCl, and 0.5 mM TCEP. The recombinant TRIM-PRYSPRY domains were concentrated to 6–17 mg/ml. Crystallization was performed using the sitting-drop vapor-diffusion method at 20 °C. Detailed crystallization conditions are given in **Supporting Information Table S3**.

### X-ray data collection and structure determination

Viable crystals of MID2, TRIM9, TRIM10, TRIM11, TRIM15, TRIM16, TRIM36, and TRIM67 were cryoprotected using mother liquor supplemented with ethylene glycol ranging from 20-24 % (v/v), MID1 crystals were cryo-protected with 25 % (v/v) glycerol before flash-freezing in liquid nitrogen. Diffraction data were collected at beamlines X06SA and X06DA of the Swiss Light Source, integrated with XDS (Kabsch, 2010) and scaled with AIMLESS (Evans, 2011). The structure of TRIM36 was solved by isomorphous replacement (single-wavelength anomalous diffraction) using crystals soaked with 10 mM mercury((o-carboxyphenyl)thio)ethyl sodium salt prior to flash-freezing. The other structures were solved by molecular replacement using PHASER (McCoy et al., 2007), with the following search models: TRIM14 (PDB: 6JBM) for MID1 and MID2, Ash2L SPRY (PDB: 4X8N) for TRIM9, TRIM7 (PDB: 6UMB) for TRIM10 and TRIM15, TRIM21 (PDB: 2IWG) for TRIM11, TRIM25 (PDB: 6FLM) for TRIM16, and TRIM9 (from this study) for TRIM67. The structures were then refined through iterative manual model building with COOT (Emsley et al., 2010) and refinement with REFMAC5 (Murshudov et al., 2011) or PHENIX (Liebschner et al., 2019). The accuracy of the final models was validated using MolProbity (Williams et al., 2018). Protein-protein interface areas were calculated using the PISA server (Krissinel and Henrick, 2007). The data collection and refinement statistics are summarized in **Supporting Table S2**.

### Differential scanning fluorimetry

The apparent melting temperature of the MID1 PRYSPRY domain was determined by differential scanning fluorimetry (DSF) using an Agilent MX3005P real-time qPCR instrument (excitation/emission filters = 492/610  nm). Measurements were performed in a 96-well plate with an assay buffer consisting of 25 mM HEPES, pH 7.5, 200 mM NaCl and a final protein concentration of 2  μM. The fluorescent dye SYPRO Orange (5000×, Invitrogen) was added at a dilution of 1:1000 (total volume of 20  μL per well). The fluorescence signal was then monitored upon temperature increase from 25 to 95 °C, at a heating rate of 3 °C/min, and the *T*_m_ value was calculated after fitting the fluorescence curves to the Boltzmann function.

## CRediT authorship contribution statement

**Rezart Zhubi:** Writing – original draft, Visualization, Investigation, Conceptualization. **Apirat Chaikuad:** Supervision, Methodology, Investigation, Conceptualization. **Christian J. Muñoz Sosa:** Investigation. **Andreas C. Joerger:** Writing – review & editing, Writing – original draft, Visualization, Supervision, Investigation, Funding acquisition, Conceptualization. **Stefan Knapp:** Writing – review & editing, Supervision, Funding acquisition, Conceptualization.

## Declaration of competing interest

The authors declare that they have no known competing financial interests or personal relationships that could have appeared to influence the work reported in this paper.

## Data Availability

The structure factors and coordinates of the TRIM PRYSPRY domains have been deposited in the Protein Data Bank (PDB) under accession codes 7QRY (MID1), 7QRZ (MID2, crystal form I), 7B2S (TRIM9), 7QS0 (TRIM10), 7QS1 (TRIM11), 7QS2 (TRIM15), 7QS3 (TRIM16), 7QS4 (TRIM36), 7QS5 (TRIM67), and 9R11 (MID2, crystal form II).
