# Supplementary material for: Structural analysis of TRIM family PRYSPRY domains and its implications for E3-ligand design
Source: J Struct Biol X. 2025 Jul 30;12:100134. doi: 10.1016/j.yjsbx.2025.100134 (PMC12355088; doi:10.1016/j.yjsbx.2025.100134)
Supplement: Supplementary Data 1 [file mmc1.pdf]

SUPPORTING INFORMATION

Structural analysis of TRIM family PRYSPRY domains and its implications for E3-ligand design

Rezart Zhubi[1,2], Apirat Chaikuad[1,2], Christian J. Muñoz Sosa[1,2], Andreas C. Joerger[1,2]*, Stefan Knapp[1,2]*

[1]Institute of Pharmaceutical Chemistry, Goethe University, Max-von-Laue-Str. 9, 60438 Frankfurt am Main, Germany

[2]Structural Genomics Consortium (SGC), Buchmann Institute for Life Sciences, Max-von-Laue-Str. 15, 60438 Frankfurt am Main, Germany

*correspondence: joerger@pharmchem.uni-frankfurt.de

or knapp@pharmchem.uni-frankfurt.de

Table S1. Sequences of TRIM PRYSPRY constructs (separate Excel file)

Table S2. X-ray data collection and refinement statistics.

Protein	**MID1**	**MID2 I**	**MID2 II**	**TRIM9**	**TRIM10**	**TRIM11**	**TRIM15**	**TRIM16**	**TRIM36**	**TRIM67**
PDB ID	7QRY	7QRZ	9R11	7B2S	7QS0	7QS1	7QS2	7QS3	7QS4	7QS5
Data Collection										
Resolution (Å)[a]	47.71-2.14<br>(2.14-2.07)	48.67-1.61<br>(1.61-1.57)	48.70-1.64<br>(1.64-1.67)	35.91-1.54<br>(1.54-1.50)	38.32-2.38<br>(2.38-2.30)	43.36-2.00<br>(2.00-1.93)	41.23-1.74<br>(1.74-1.70)	45.69-1.81<br>(1.81-1.75)	46.53-2.33<br>(2.33-2.25)	47.87-1.69<br>(1.69-1.65)
Space group	P1	$C222_1$	$P2_12_12_1$	C2	$P3_1$	$P2_1$	$P4_12_12$	$P4_12_12$	$P2_12_12_1$	$I4_1$
Cell parameters:<br>a, b, c (Å); α, β, γ (°)	a=38.86,<br>b=51.76,<br>c=80.83;<br>α=103.76,<br>β=92.64,<br>γ= 94.32	a=63.92,<br>b=75.08,<br>c=78.19;<br>α=β=γ=90	a=53.41,<br>b=60.92,<br>c=118.59;<br>α=β=γ=90	a=61.62,<br>b=45.28,<br>c=57.66;<br>α=γ=90,<br>β=106.96	a=b=65.99,<br>c=103.31;<br>α=β=90,<br>γ=120	a=65.64,<br>b=42.02,<br>c=68.58;<br>α=γ=90,<br>β=99.44	a=b=54.76,<br>c=125.26;<br>α=β=γ=90	a=b=55.73,<br>c=159.60;<br>α=β=γ=90	a=91.74,<br>b=93.06,<br>c=103.85;<br>α=β=γ=90	a=b=135.39,<br>c=35.80;<br>α=β=γ=90
Molecules/AU	4	1	2	1	3	2	1	1	4	2
No. unique reflections	35,045	26,626	48,227	24,080	22,199	27,861	21,814	26,339	42,900	39,503
Completeness (%)[a]	95.0 (94.6)	99.9 (99.8)	99.9 (100)	98.6 (97.6)	99.2 (100)	99.2 (99.4)	100 (100)	99.9 (99.9)	100 (100)	99.9 (100)
$I/\sigma(I)^a$	5.5 (2.0)	20.8 (2.8)	14.6 (1.9)	33.9 (19.5)	3.4 (1.8)	12.3 (2.0)	15.7 (2.2)	16.3 (2.3)	16.9 (2.0)	17.7 (2.4)
R_{merge}^a	0.128<br>(0.545)	0.063<br>(0.93)	0.070<br>(1.054)	0.033<br>(0.062)	0.095<br>(0.378)	0.071<br>(0.841)	0.118<br>(1.221)	0.061<br>(1.060)	0.066<br>(0.983)	0.065<br>(0.999)
CC1/2[a]	0.999<br>(0.867)	1.000<br>(0.905)	0.999<br>(0.812)	0.998<br>(0.995)	0.997<br>(0.986)	0.998<br>(0.794)	0.999<br>(0.767)	0.999<br>(0.915)	0.999<br>(0.861)	0.999<br>(0.851)
Multiplicity[a]	4.0 (4.0)	11.2 (11.5)	9.0 (9.2)	5.8 (5.4)	8.7 (9.0)	5.9 (6.1)	12.2 (12.5)	9.0 (9.1)	9.3 (9.4)	10.6 (10.8)
Refinement										
No. of atoms in refinement	5259	1380	2920	1406	4223	2836	1343	1625	6118	2597
No. of waters in refinement	117	134	297	239	76	131	102	149	97	221
R_{cryst}^b	0.2269	0.1624	0.1553	0.1416	0.2038	0.1683	0.1688	0.1630	0.2026	0.1548
R_{free}^b	0.2726	0.1921	0.2057	0.1738	0.2561	0.2049	0.1990	0.1979	0.2449	0.1771
Overall B factor (Å^2)	49.3	19.8	31.0	11.3	43.9	36.7	18.4	32.4	56.6	24.7
RMSD bond (Å)	0.010	0.016	0.006	0.016	0.011	0.015	0.015	0.015	0.012	0.016
RMSD angle (°)	1.29	1.65	0.83	1.59	1.23	1.46	1.57	1.45	1.25	1.51
Ramachandran Favored (%)	94.1	95.8	96.6	98.2	96.9	98.6	97.7	96.9	97.0	96.6
Ramachandran Outliers (%)	0.0	0.0	0.3	0.0	0.0	0.0	0.0	0.0	0.14	0.0

[a]Values in parentheses are for the highest-resolution shell.
[b]R_{cryst} and $R_{free} = \sum||F_{obs}| - |F_{calc}||/\sum|F_{obs}|$, where R_{free} was calculated with 5% of the reflections chosen at random and not used in the refinement.

Table S3. Crystallization conditions of TRIM PRYSPRY domains

Protein	Protein concentration (mg/ml)[a]	Crystallization buffer
MID1	11.9	1.1 M sodium citrate, 0.1 M HEPES, 7.5
MID2-I	14.9	0.2 M ammonium sulfate, 18% high molecular weight PEG smears, 0.1 M ADA, pH 6.5
MID2-II	9.0	7% PEG 6000, 5% MPD, 0.1 M HEPES, pH 7.0
TRIM9	15.0	0.2 M ammonium sulfate, 30% PEG 8000
TRIM10	16.7	25% ethylene glycol, pH 7.5
TRIM11	14.0	20% PEG 3350, 10% ethylene glycol, 0.2 M sodium sulfate, pH 7.5
TRIM15	15.0	0.2 M sodium iodide, 20% PEG 3350, 10% ethylene glycol, 0.1 M bis-tris-propane, pH 6.5
TRIM16	6.0	0.2 M sodium bromide, 20% PEG 3350, 10% ethylene glycol, 0.1 M bis-tris-propane, pH 6.5
TRIM36	13.1	24% PEG 3350, 0.15 M sodium citrate, pH 7.5
TRIM67	13.2	20% high molecular weight PEG smears, 0.1 M acetate, pH 4.5

[a]Protein buffer: 20 mM HEPES pH 7.5, 100 mM NaCl, and 0.5 mM TCEP.

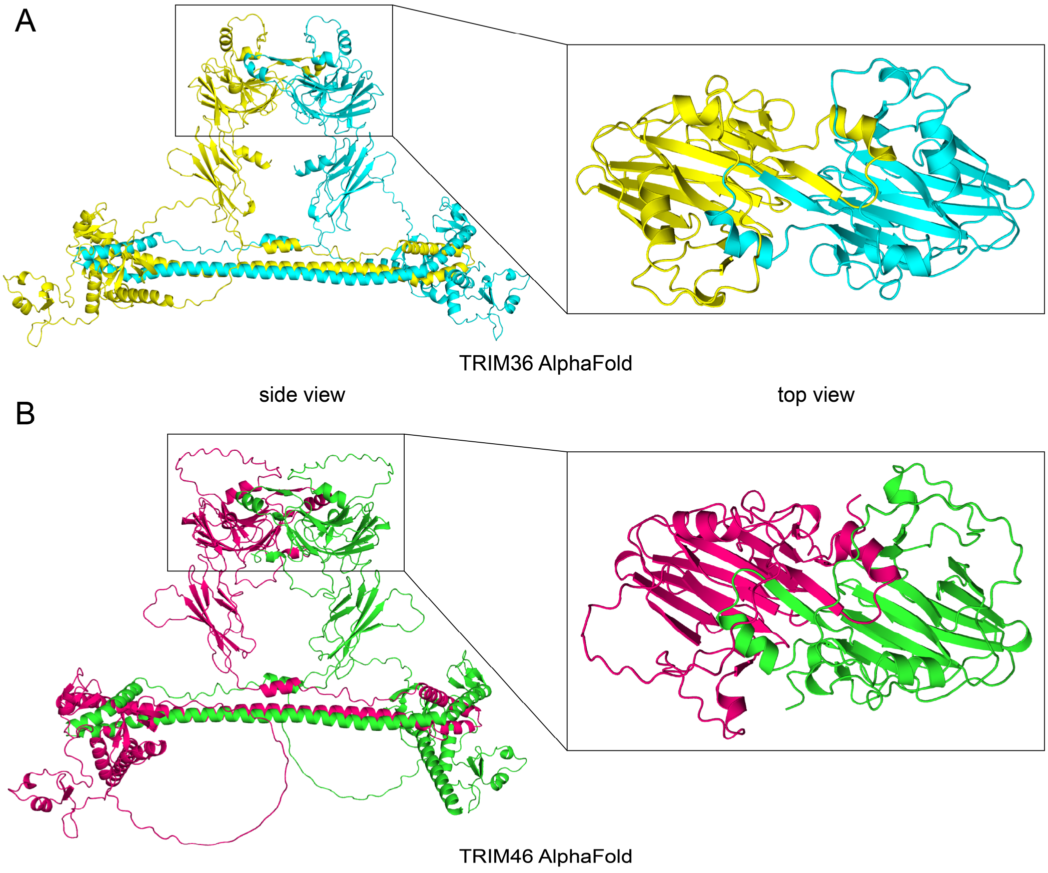

Figure S1. AlphaFold3 models of the dimers of full-length TRIM36 (A) and TRIM46 (B).
